# Oral Delivery of Nanoparticles Carrying Ancestral Uricase Enzyme Protects against Hyperuricemia in Knockout Mice

**DOI:** 10.1021/acs.biomac.2c01388

**Published:** 2023-04-26

**Authors:** Lily Tran, Soumen Das, Liangjun Zhao, M.G. Finn, Eric A. Gaucher

**Affiliations:** † Department of Biology, Center for Diagnostics and Therapeutics, Georgia State University, Atlanta, Georgia 30303, United States; ‡ School of Chemistry and Biochemistry, Georgia Institute of Technology, Atlanta, Georgia 30306, United States; § School of Biological Sciences, Georgia Institute of Technology, Atlanta, Georgia 30306, United States

## Abstract

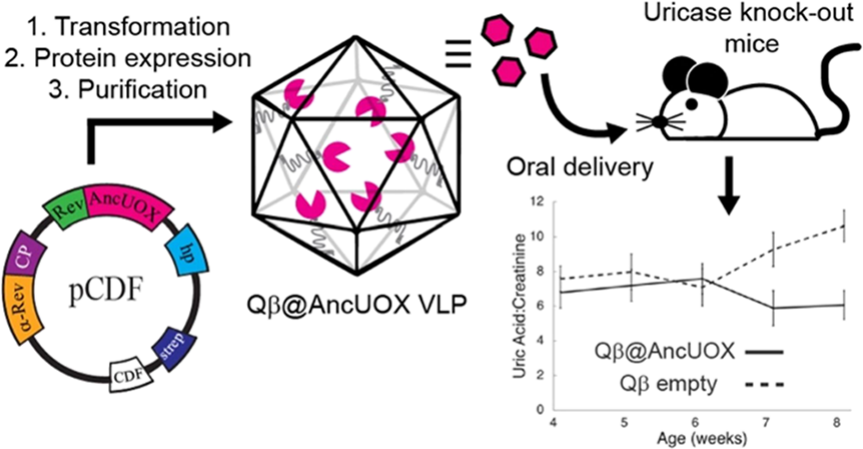

The therapeutic value of delivering recombinant uricase to human patients has been appreciated for decades. The development of therapeutic uricases has been hampered by the fact that humans do not encode an endogenous uricase and therefore most recombinant forms of the protein are recognized as foreign by the immune system and are therefore highly immunogenic. In order to both shield and stabilize the active enzyme, we encapsulated a functional ancestral uricase in recombinant, noninfectious Qβ capsid nanoparticles and characterized its catalytic activity. Oral delivery of the nanoparticles moderated key symptoms of kidney dysfunction in uricase-knockout mice by lowering uric acid levels. Histological kidney samples of the treated mice suggest that delivery of recombinant uricase had a protective effect against the destructive effects of uric acid that lead to renal failure caused by hyperuricemia.

## Introduction

Uric acid is a small molecule generated mostly by purine metabolism and is subsequently excreted by host organisms after it is oxidized to soluble molecules. The concentrations of endogenous uric acid levels are controlled by enzymes upstream and downstream of uric acid in its metabolic pathway, as well as membrane transporters of the small molecule.^1,2^ This control is important because uric acid becomes insoluble and forms crystalloids at concentrations near 450 μM, which can quickly cause renal failure in mammals.^3^ Even sustained moderate levels of uric acid can also cause crystals to accumulate in avascular tissue (e.g., cartilage, tendons, and ligaments) and lead to long-term complications, such as gout.^4^ Apes (including humans) are unusual among mammals in the control of uric acid because they lack a functional form of the enzyme uricase that would otherwise oxidize uric acid.^5^ Further, the uric acid membrane transporters in ape kidneys have evolved to reabsorb most of the uric acid from urine at normal physiological conditions and return it back to the blood, while non-apes excrete most of the small molecule as waste.^6^

Regardless of the evolutionary cause of uricase deactivation in apes, modern humans can suffer from an inability to properly manage endogenous uric acid levels. Accordingly, a great deal of effort over many years in both academia and industry has gone into the development of recombinant uricases and inhibitors of uric acid transporters. However, a safe and effective therapeutic uricase remains elusive. Much of the therapeutic uricase development has focused on shielding the recombinant uricase protein from the immune system. Some of the earliest formulations employed PEGylation of the uricase protein, but these had limited success. The launch of Krystexxa (a PEGylated version of a Pig-Baboon Chimeric, or PBC, uricase) nearly one decade ago has become a textbook case of caution. Most human subjects exhibited strong immune responses to the drug^7−9^ and the FDA issued a black-box warning, making most doctors reluctant to prescribe and administer it. A number of uricase variants or formulations are currently being developed to address this unmet need in the gout/hyperuricemia market. For example, Krystexxa is being reformulated with an improved PEGylated moiety by 3SBIO (Shenyang, China), Selecta Biosciences (MA, USA) has encapsulated a PEGylated *Candida* uricase in viral particles to prevent an immune response in human patients^10,11^ and China Pharmaceutical University has engineered a uricase that essentially uses the ancestral sequence reconstruction approach to reactivate the human gene into a functional enzyme^12,13^ Horizon Therapeutics (Dublin, IE) is currently co-administering the immune suppressor methotrexate along with PEGylated PBC uricase in clinical trials and demonstrated that patients have a lower immune response with this combination.^14^ In 2019, an academic research group reported the engineering of a uricase from the bacteria *Arthrobacter* that showed greater resistance to general protease digestion compared to other uricases.^15^ In 2020, Fagan Biomedical Inc. reported its development of a PEGylated canine uricase that has high bioavailability in monkeys.^16^ Lastly, an academic group has used Bmk9 peptides to form nanoparticles that link uricase to its surface for the treatment of chronic hyperuricemia.^17^

All of the above strategies involve injection of the therapeutic agent; oral delivery of active uricase would obviously be preferred from the perspective of patient compliance and convenience and because the lower intestine is now known to play an important role in lowering serum uric acid levels.^18^ One recent approach used *Staphylococcus* engineered to export uricase out of the bacterial cell. Delivery of the bacterium to rats by oral gavage resulted in colonization of the intestines, the release of uricase, and lowering of uric acid levels in the intestines.^19^*Candida* uricase has also been engineered to be hyperstable against the pH environment of the digestive tract. Animal studies have demonstrated that the oral administration of this uricase can lower serum uric acid levels^20,21^ and clinical studies in humans are currently being conducted by Allena Pharmaceuticals (MA, USA).

We have previously demonstrated that ancestral uricases can have superior stability compared to unmodified *Candida* and PBC uricases under conditions that simulate the mammalian gastrointestinal tract.^22^ Here, we combine this advantage with the ability of virus-like particle (VLP) capsids to conveniently encapsulate and stabilize enzymes.^23,24^ Thus, a highly active ancestral uricase recombinantly produced with, and entrained within, Qβ VLPs were orally delivered to uricase-knockout mice. These agents demonstrated promising therapeutic potential by lowering endogenous uric acid levels in a dose-dependent and extended fashion.

## Materials and Methods

### Cloning, Production, and Purification of Enzyme-Packaged Nanoparticles

Bicistronic plasmids coding for both Qβ capsid protein and Rev-tagged AncUOX in the pCDF-1b parent vector, as previously described.^23^ All constructs were verified by sequencing before expression experiments. *E. coli* BL21 (DE3) (Biogen) cells harboring the appropriate plasmids were grown in SOB (Amresco) supplemented with 20 mM magnesium sulfate and 50 μg/mL streptomycin. The starter cultures were grown overnight at 37 °C and used to inoculate larger expression cultures. The expression was induced with 1 mM IPTG when OD_600_ reached about 1.0, and the induced culture was kept at room temperature for overnight expression. Cells were harvested by centrifugation in a JA-10 rotor at 6000 rpm, and the pellets were either processed immediately or stored at −80 °C. The cell lysate was prepared by re-suspending the cell pellet with 100 mL of 100 mM potassium phosphate (KPhos) buffer (pH 7.0) and sonicating at 30 W for 10 min with 5 s bursts and 5 s intervals. Cell debris was pelleted in a JA-17 rotor at 14,000 rpm, and 0.265 gm/mL ammonium sulfate was added to the supernatant to precipitate the VLPs. The crude virus-like particle pellet from precipitation was re-suspended in 6 mL of 100 mM KPhos buffer (pH 7.0). Organic extraction with 1:1 n-butanol:chloroform was performed to remove lipids and other cellular debris from VLPs. The aqueous layer containing VLPs were further purified by sucrose density ultracentrifugation (10–40% w/v). Particles were pelleted out by ultracentrifugation in a 70Ti rotor (Beckman) at 68,000 rpm for 2 h. The resulted particle is denoted as Qβ@AncUOX.

### Characterization of Enzyme-Packaged Nanoparticles

The purity of synthesized Qβ@AncUOX nanoparticles was assessed by isocratic size exclusion chromatography with a Superose 6 column on an FPLC instrument. Non-aggregated Qβ particles were eluted at about 12 mL after the void volume-associated peaks. Microfluidic denaturing gel electrophoresis (Agilent Bioanalyzer 2100, Protein 80 chip) was used to analyze the average number of enzymes packaged inside the particles, determined by normalizing the integrated intensity of coat protein and cargo protein bands by their respective molecular weights, assuming no differences in staining. A factor of 180 was used to adjust the number of cargo proteins loaded per nanoparticle as each capsid is composed of 180 copies of the coat protein. The overall protein concentration was determined with Coomassie Plus Protein Reagent (Pierce) according to the manufacturer’s instructions.

### Enzyme Activity Assay and Thermostability Assay

All experiments were run in triplicate with individually purified particles. Qβ@AncUOX enzyme activity was measured by monitoring the conversion of uric acid to allantoin using the UV absorbance at 293 nm in Evolution 200 UV–vis spectrophotometer equipped with Peltier. The molar extinction coefficient at 293 nm of uric acid (13.2 mM^–1^ cm^–1^) was determined by a standard curve. For determinations of kinetic parameters at 37 °C, first 995 μL of 0–100 μM substrate in 100 mM potassium phosphate buffer (pH 7.0) incubated at that temperature for 5 min. Then, 5 μL of a 4 mg/mL solution of Qβ@AncUOX was diluted 200-fold and read immediately. A Michaelis–Menten non-linear fit was used to obtain *K*_M_ and *k*_cat_ values.

To determine the thermal half-life of the Qβ-packaged AncUOX enzymes at 37 °C, a 4 mg/mL solution of Qβ@AncUOX was incubated at 37 °C water bath. At each time interval, the enzyme was diluted 200-fold and the activity was measured as above. Activity measurements were plotted vs time and a first order exponential decay nonlinear fit was used to obtain the half-life value (Figure S1).

### Oral Delivery of Nanoparticles to Knockout Mice

Uricase-knockout (KO) mice (stock #002223, B6;129S7-*Uox^tm1Bay^*/J^25^) purchased from Jackson Laboratory (Bar Harbor, ME, USA) were bred in-house and given *ad libitum* access to 0.15 g/L allopurinol via water (Sigma-Aldrich, St. Louis, MO, USA). Experiments began when KO pups were born and randomly assigned to two groups: Qβ@AncUOX (Qβ vector containing ancestral uricase) and control (empty Qβ vector) (*n* = 8, four females and four males in each group). The mother’s access to allopurinol water was ceased when the pups were two weeks old (allopurinol is passed to pups through breast milk) to ensure that the mice were in a distressed state prior to chronic kidney malfunction. The pups were weaned at three weeks of age and administered 0.5 mg Qβ@AncUOX (containing approximately 0.05 mg of uricase) or empty Qβ particles by oral gavage twice daily with a 1-h gap between the two doses, for a total of four weeks. The mice showed no physiological symptoms from being gavaged. Urine was collected weekly within a 2-h window prior to the oral gavage. Uric acid and creatinine levels were quantified with a Uric Acid/Uricase Assay Kit and Creatinine Assay Kit, respectively, from Cell BioLabs, Inc. (San Diego, CA, USA) according to the manufacturer’s protocol. Each urine sample was assayed in triplicate for uric acid and duplicate for creatinine. Mice were euthanized at eight weeks of age, and the kidneys were immediately harvested and fixed in 100% ethanol prior to being sent to IDEXX BioAnalytics (Columbia, MO, USA) for histopathological analyses. All animal experiments were approved by the Institutional Animal Care and Use Committee (IACUC protocol# A21013 approved at Georgia State University).

### Kidney Histology

Fixed kidneys were trimmed by longitudinal bisection and processed for paraffin infiltration. The samples were blocked, sectioned, and stained with hematoxylin and eosin (H&E) and permanently cover-slipped (conducted at IDEXX BioAnalytics by trained pathologists).

## Results and Discussion

**Figure 1:**
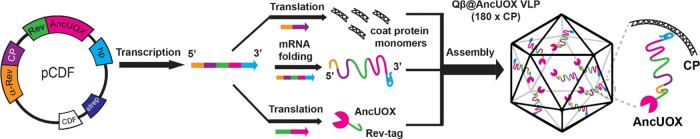
Generation of Qβ nanoparticles containing ancestral uricase. A bicistronic expression vector with compatible T7 promoters was used to drive expression of capsid protein, Rev-tagged AncUOX, and bifunctional mRNA. The Rev-tag binds to the α-Rev aptamer and Qβ genome packaging hairpin binds to the interior of the CP monomers, directing the Rev-tagged AncUOX packaged into the interior of the Qβ nanoparticle.

**Figure 2:**
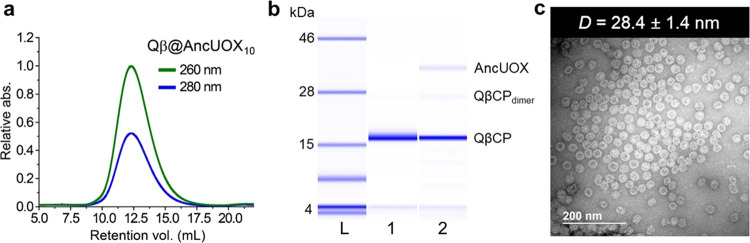
Characterization of Qβ@AncUOX nanoparticles. (a) Size-exclusion FPLC showing the intact nature of the VLPs. (b) Microfluidic electrophoresis (Bioanalyzer). Lanes: L = MW ladder, 1 = Qβ-wt, 2 = Qβ@AncUOX_10_. (QβCP_dimer_ = noncovalent coat protein dimer resulting from incomplete denaturation). (c) Negative-stain TEM image of Qβ@AncUOX nanoparticles (*D* = diameter).

Uricase is a 33 kDa monomer protein but functions as a non-covalent homotetramer to catalyze the oxidation of insoluble uric acid to soluble 5-hydroxyisourate. An ancestral uricase (AncUOX) sequence was modified by the installation of an N-terminal Rev peptide tag to facilitate VLP packaging.^26^ Simultaneous expression of the modified AncUOX with Qβ capsid protein and untranslated mRNA bearing a Rev-binding aptamer at one end and a VLP-binding aptamer at the other resulted in the production of VLPs containing packaged enzyme (designated Qβ@AncUOX_*n*_, *n* = packaging number per VLP, Figure 1). These particles were isolated, purified, and characterized by methods standard for VLPs (Figure 2) and were found to contain an average of 10 copies of the AncUOX enzyme per capsid (Figure 2b). Size exclusion chromatography (Figure 2a) and transmission electron microscopy (TEM, Figure 2c) showed the particles to be intact, non-aggregated, and the same size as native Qβ-wt VLPs.

**Figure 3:**
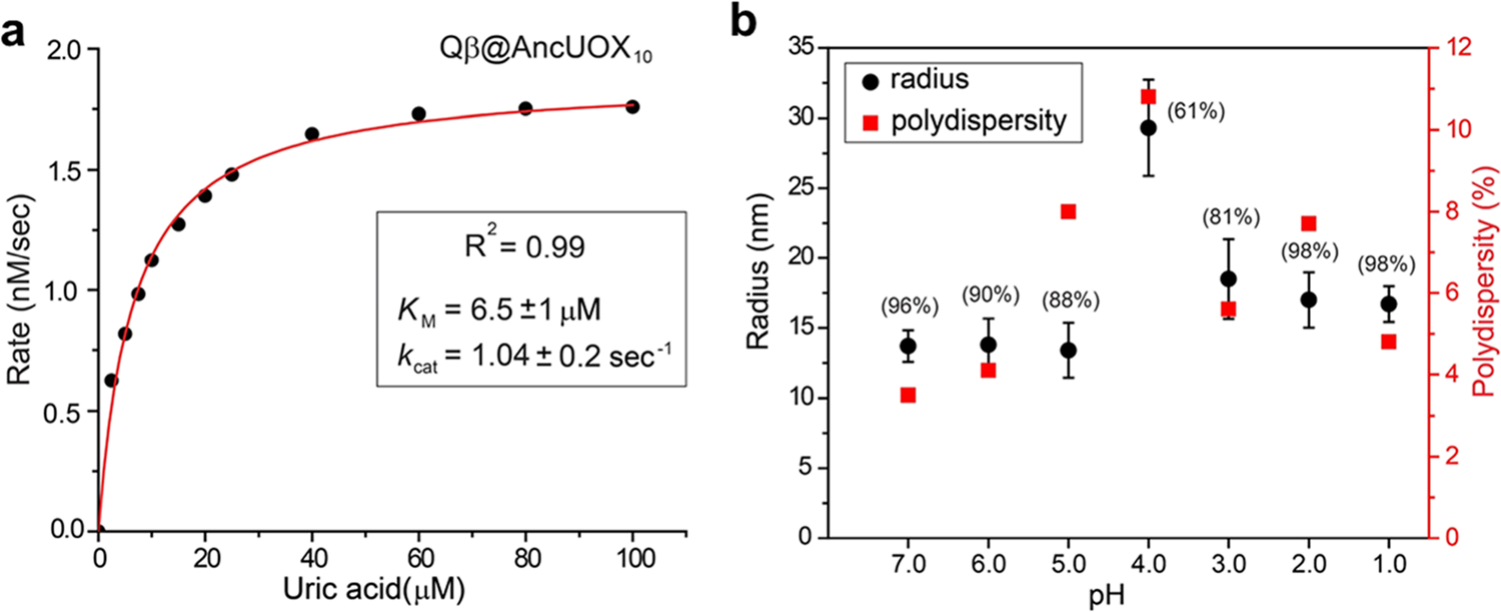
(a) Michaelis–Menten kinetic analysis of Qβ@AncUOX reactivity. (b) Stability test of Qβ@AncUOX nanoparticles at low pH: radii and polydispersity as determined by dynamic light scattering for Qβ@AncUOX particles incubated for 24 h at the indicated pH (100 mM glycine-HCl buffer). Values in parentheses report the concentration of particles after incubation relative to the concentration before incubation.

The kinetic activity of Qβ@AncUOX was characterized by monitoring the disappearance of substrate uric acid. Michaelis–Menten treatment of initial-rate data (Figure 3a) gave a catalytic efficiency of approximately 2 × 10^5^ M^–1^ s^–1^ per enzyme tetramer, similar to the approximately 5 × 10^5^ M^–1^ s^–1^ value reported for unpackaged enzyme.^22^ Thus, the urate/uric acid is able to freely diffuse through the capsid shell and the catalytic power of the ancestral enzyme is not affected by sequestration in the VLP interior. Packaged AncUOX enzymes were found to lose activity upon incubation at 37 °C with a half-life of 4 h (Figure S1), compared to <5 min for the free (unpackaged) enzyme. Kinetic characterization of one-year-old samples of Qβ@AncUOX nanoparticles stored at 4 °C showed nearly no change in catalytic efficiency of the packaged enzyme, an identical enzymatic observation as the unpackaged free-enzyme at the same temperature for the same period of time.

To survey potential limitations in gastrointestinal stability, Qβ@AncUOX nanoparticles (25 nM) were incubated in glycine–HCl buffer (100 mM) at pH values from 1.0 to 7.0, each for 24 h at room temperature and characterized by dynamic light scattering (DLS) and TEM. The former (summarized in Figure 3b) showed pH-dependent aggregation near the anticipated isoelectric point of the particles, some change in particle shape at pH 1–2 but no decomposition or loss by precipitation at any pH. These results are supported by TEM images (Figure S2). Therefore, the Qβ-nanoparticle encapsulation stabilizes encapsulated uricase at low pH, as previously observed for a near-infrared fluorescence protein.^23^

**Figure 4:**
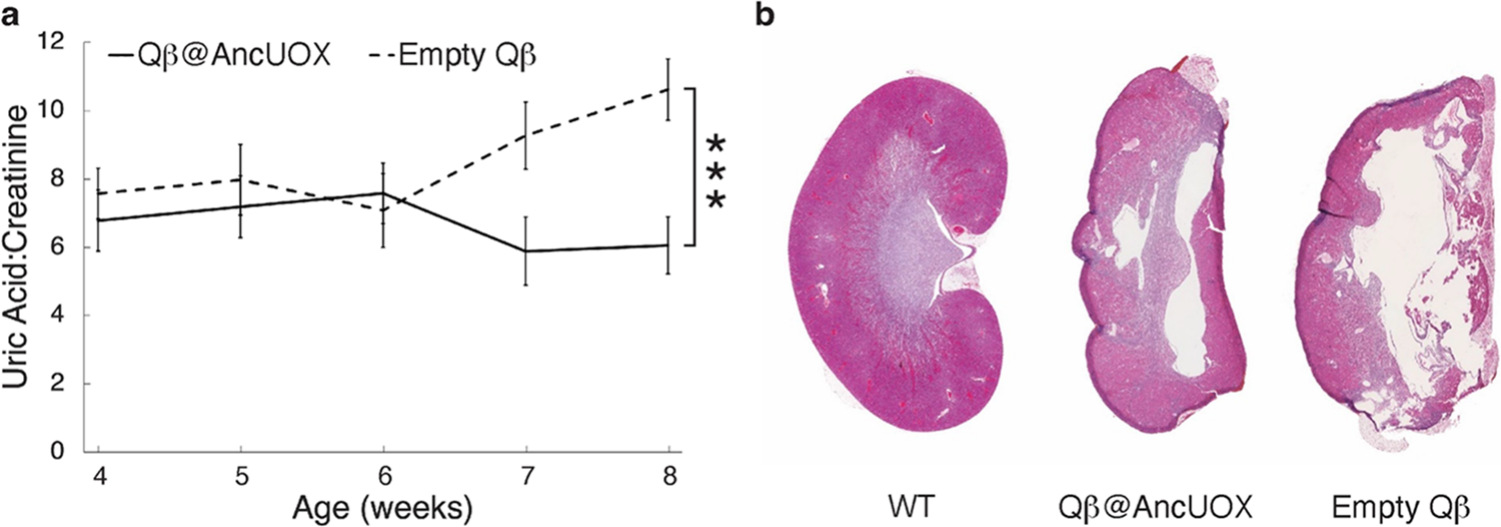
Oral gavage treatment using Qβ@AncUOX in mice. Uricase knock-out mice were administered AncUOX encapsulated in a Qβ capsid nanoparticle (Qβ@AncUOX) or the capsid alone, termed empty Qβ, by oral gavage twice daily for four weeks starting at three weeks of age, weaned off allopurinol at two weeks of age to ensure mice were in a distressed state prior to chronic kidney disease. (a) The ratio of urine uric acid to urine creatinine was monitored once per week for six weeks (ages 3–8 weeks). Data are presented as mean ± SEM for Qβ@AncUOX (solid line) and empty Qβ (dashed line), *n* = 8 (four females and four males per group), *** denotes *p* = 0.001 (Student’s *t*-test in SPSS Statistics). (b) Representative H&E stained kidney sections from mice treated with the indicated nanoparticles, as well as from an untreated wild-type mouse (expressing endogenous uricase) of the same age.

Qβ@AncUOX nanoparticles were orally delivered to uricase-knockout mice by gavage twice daily for four weeks. As seen in Figure 4a, this treatment significantly prevented the buildup of uric acid in the urine of uricase-knockout mice even one-week after the end of the treatment period compared to the nontreated group (treatment ended at age of 7 weeks, last samples were collected at age of 8 weeks). Conversely, oral delivery of empty Qβ vector was not able to prevent an increase in uric acid levels associated with hyperuricemia in aging uricase-knockout mice.

To assess whether Qβ@AncUOX can prevent kidney damage, we analyzed mouse kidneys post-treatment. Figure 4B shows representative kidneys of wildtype (normal uricase), and uricase-knockout with and without treatment (kidney images for all sixteen mice shown in Figure S3). Although the gross morphology of the latter two from knockout mice appeared to be similar, reflecting severe renal damage prior to treatment, the mice that received the VLP-packaged uricase had fewer tubular cysts, less serve hydronephrosis and tubular degeneration, and fewer interstitial mononuclear infiltrates than the control VLP-only treated group. Most notably, the Qβ@AncUOX treated mice displayed substantially less parenchymal collapse.

We have demonstrated that a recombinant ancestral uricase packaged in Qβ nanoparticles and orally delivered to uricase-knockout mice is able to prevent the buildup of endogenous uric acid levels and suppress some of the renal damage caused by hyperuricemia during a limited treatment regime. The use of virus-like particles as oral vaccine candidates is well known, and a few accounts have described or inferred remarkable stability of different VLPs toward the degradation in the GI tract^27−29^ While we have not directly analyzed the fate of Qβ VLPs after oral delivery, the retention of significant uricase activity, presumably in the intestine, suggests that this particle also resists digestive enzymes and acidic hydrolysis, and imparts hydrolytic stability to the packaged enzyme as well.

Uric acid plays a role in metabolic disorders, hypertension, gout, and also in cancer.^30^ The oral delivery of uricase to treat hyperuricemia would circumvent most of the immunological concerns associated with injecting a recombinant uricase into an animal model or human patient. Most importantly, the ability to control serum uric acid levels via the gastrointestinal (GI) tract over long periods would substantially alter the prospects for the treatment of gout and the management of hyperuricemia in the future.

## Conclusions

In summary, we have shown that oral delivery of nanoparticles containing functional uricase enzyme is able to lower uric acid levels in the urine of uricase-knockout mice. The lowering of endogenous uric acid levels is correlated with reduced kidney dysfunction associated with hyperuricemia in these uricase-knockout mice. Our results support a growing body of evidence that oral delivery of uricase can provide therapeutic value to individuals suffering from the devasting effects of hyperuricemia. Future studies will include dose-dependent administration of our nanoparticles to determine how uric acid levels in the blood of uricase-knockout mice may be controlled by the presence of uricase in the GI tract.

## Supporting Information Available

Activity decay of the Qβ@AncUOX nanoparticles, DLS and TEM images of the Qβ@AncUOX nanoparticles, images of kidney slices from all 16 experimental mice (PDF)
