# Supplementary material for: Oral Delivery of Nanoparticles Carrying Ancestral Uricase Enzyme Protects against Hyperuricemia in Knockout Mice
Source: Biomacromolecules. 2023 Apr 26;24(5):2003–8. doi: 10.1021/acs.biomac.2c01388 (PMC10170503; doi:10.1021/acs.biomac.2c01388)
Supplement: Supplementary file 1 — bm2c01388_si_001.pdf [file bm2c01388_si_001.pdf]

Supporting Information

Oral delivery of nanoparticles carrying ancestral uricase enzyme protects against hyperuricemia in knockout mice

Lily Tran[1], Soumen Das[2], Liangjun Zhao[2], M.G. Finn[2,3], Eric A. Gaucher[1*]

1. Department of Biology, Center for Diagnostics and Therapeutics, Georgia State University, Atlanta, GA U.S.A. 30303

2. School of Chemistry and Biochemistry, Georgia Institute of Technology, Atlanta, GA U.S.A. 30306

3. School of Biological Sciences, Georgia Institute of Technology, Atlanta, GA U.S.A. 30306

*Corresponding author information

100 Piedmont Ave

Atlanta, GA 30303

Tel: 404-413-5432

Email: egaucher@gsu.edu

This file includes Supporting Figures S1, S2 and S3.

Supplemental Figure S1

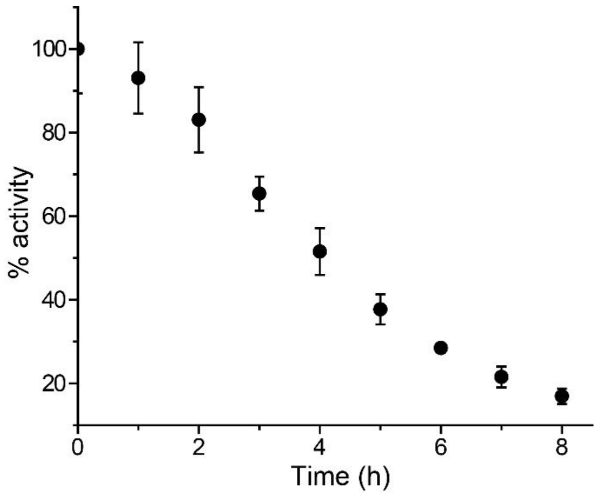

Figure S1. Activity decay of the Qβ@AncUOX particles when incubated at 37°C.

Supplemental Figure S2

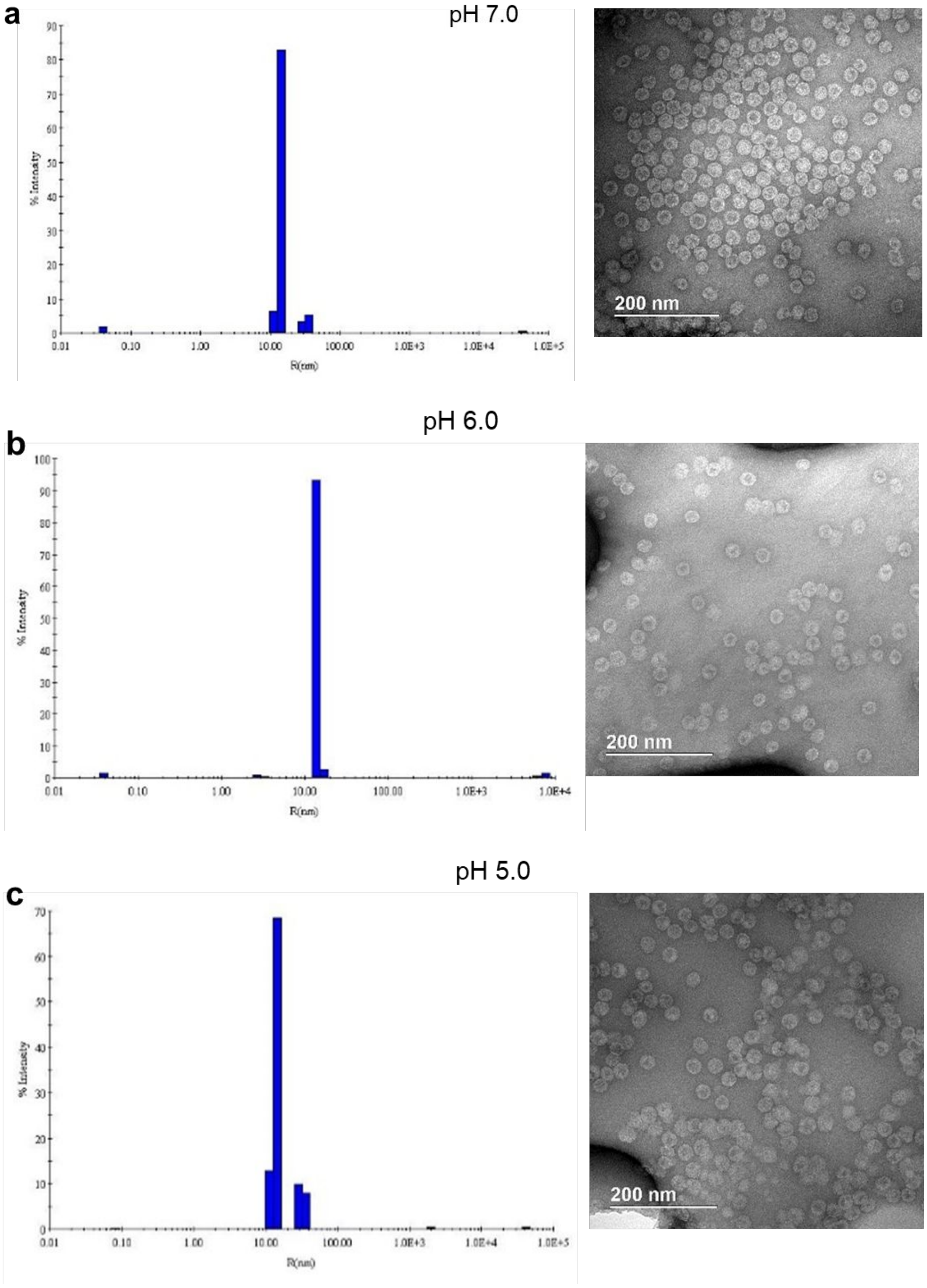

d pH 4.0

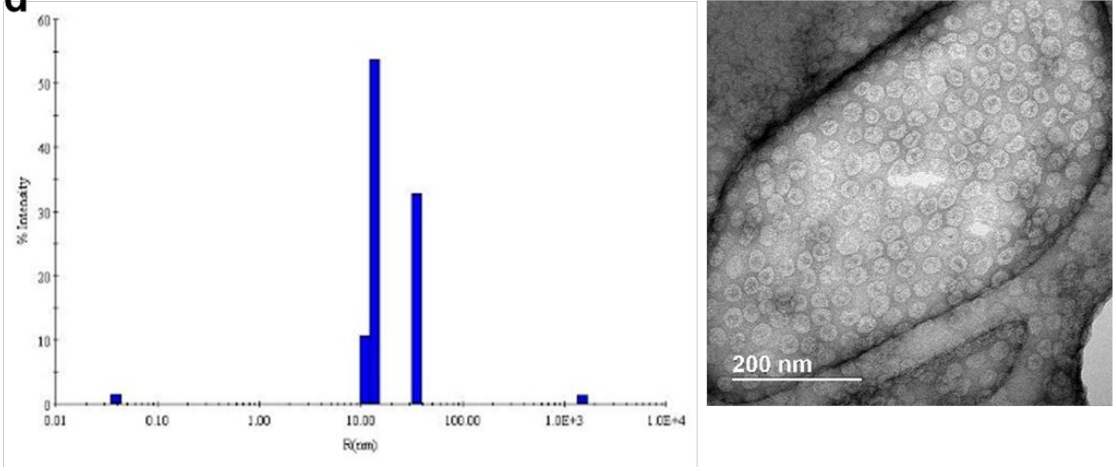

e pH 3.0

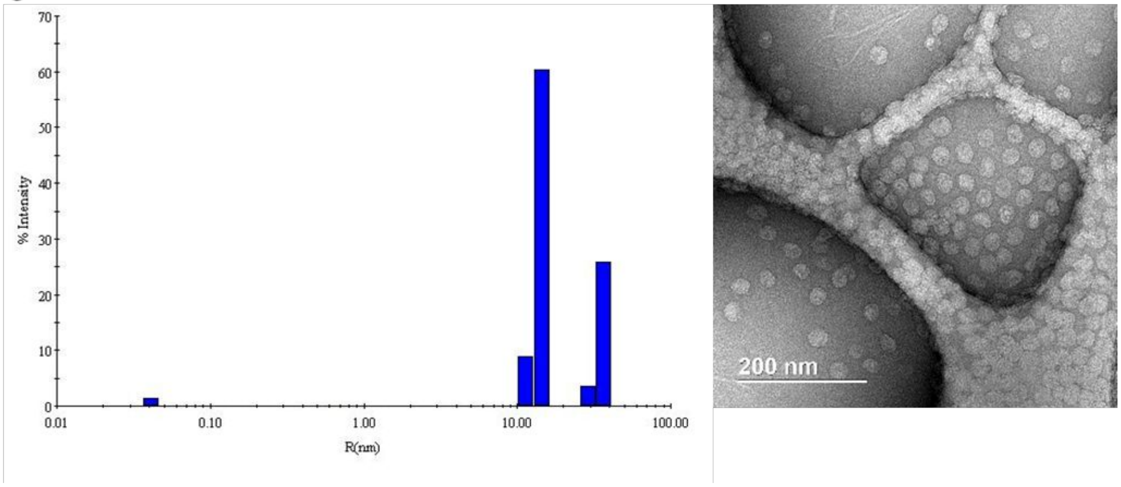

f pH 2.0

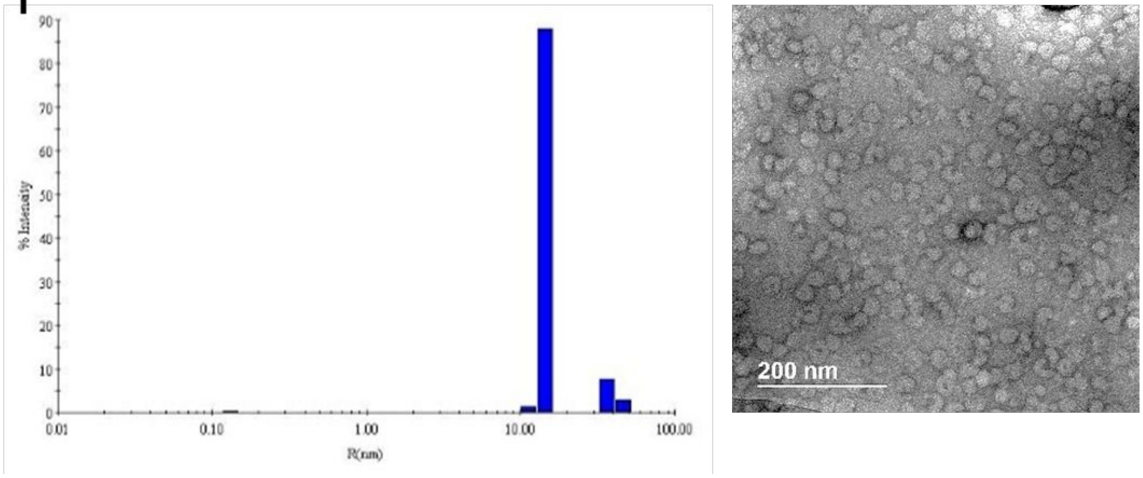

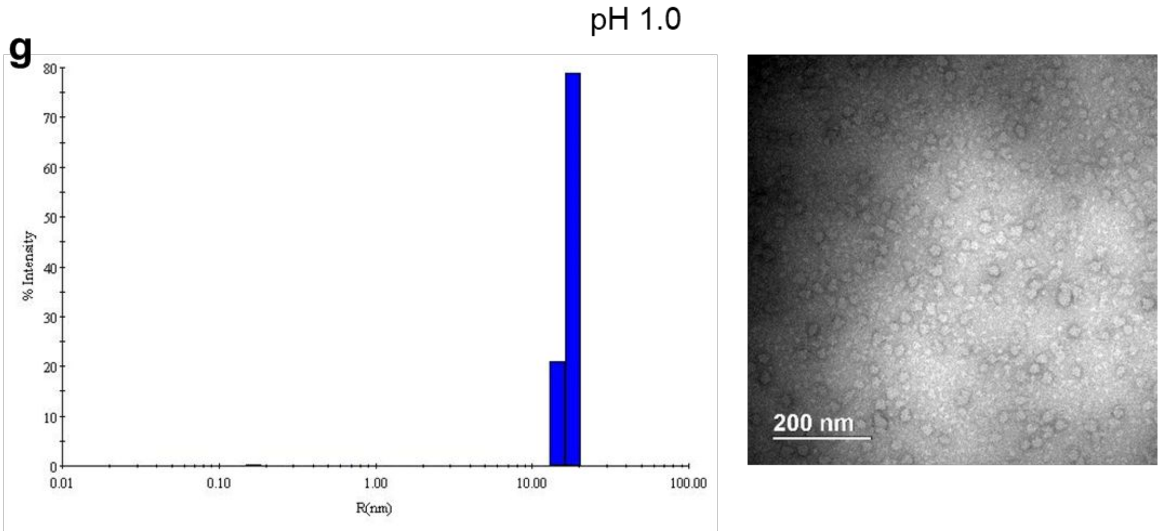

Figure S2. DLS and TEM images of the Qβ@AncUOX nanoparticles after incubation at different pH for 24 h.

Supplemental Figure S3

a

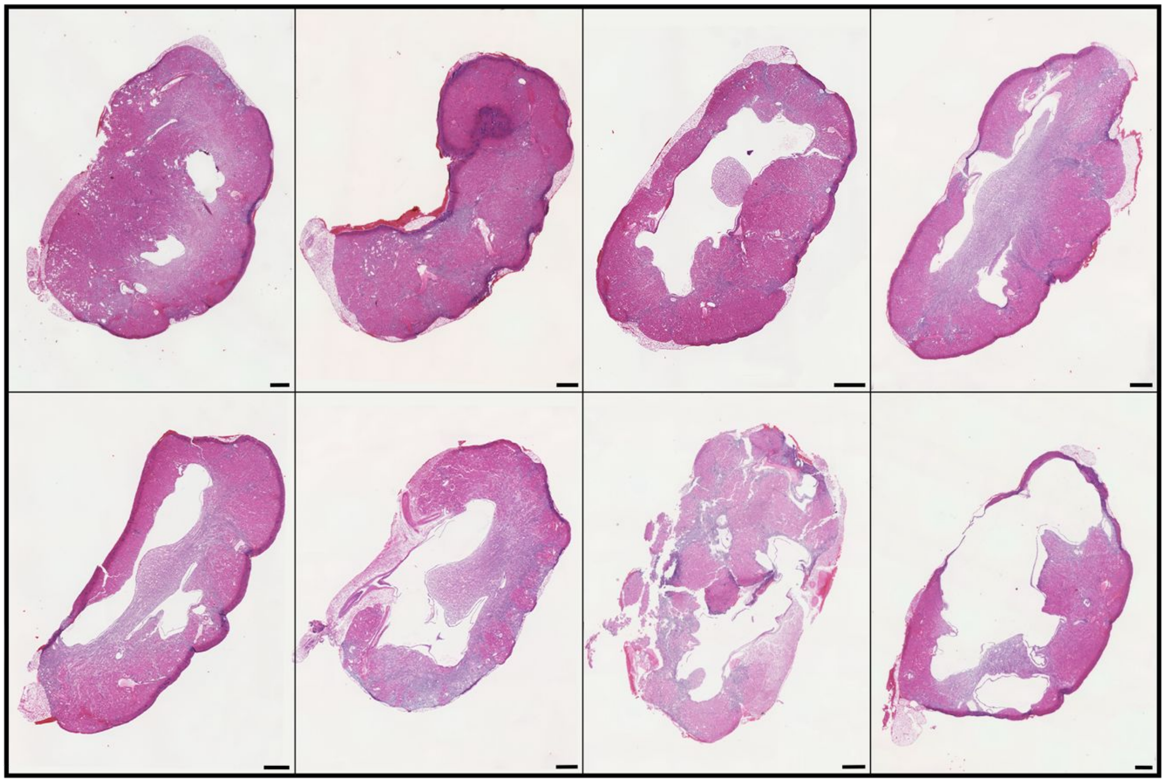

b

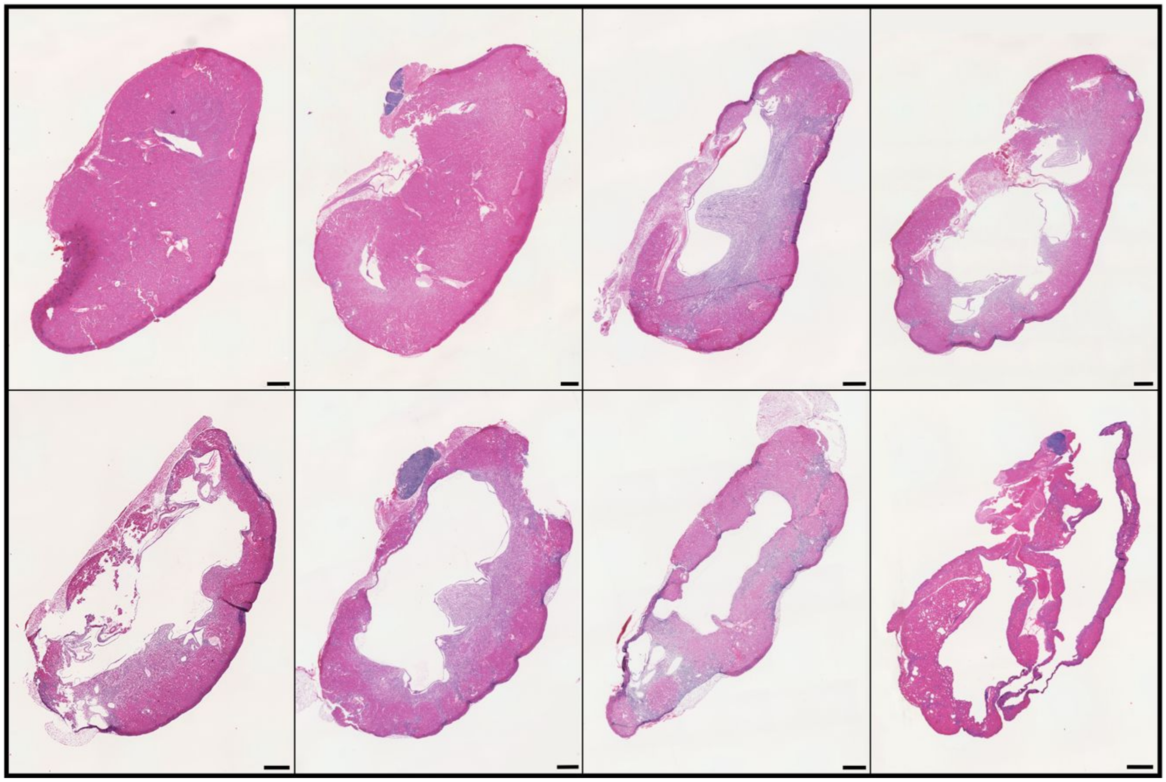

Figure S3. Kidney histology images from all 16 mice utilized in the study. Uricase knock-out mice were administered AncUOX encapsulated in a Qβ capsid nanoparticle, termed Qβ@AncUOX, or the capsid alone, termed empty Qβ, by oral gavage twice daily for four weeks starting at three weeks of age, weaned off allopurinol at two weeks of age to ensure mice were in a diseased state prior to chronic kidney disease. Kidney sections for eight mice each in (a) Qβ@AncUOX group and (b) empty Qβ control group. Kidneys were harvested and stained with hematoxylin and eosin for histological analysis by IDEXX Laboratories. Scale bar = 500 micrometers.
